# Ophthalmia Neonatorum; a Pathologic Anachronism1An address delivered by invitation at the twentieth annual meeting of the American Association of Obstetricians and Gynecologists, held at Detroit, September, 17-19 1907

**Published:** 1908-01

**Authors:** F. Park Lewis

**Affiliations:** Buffalo, N. Y.


### Ophthalmia Neonatorum; a Pathologic Anachronism.[1]

By F. PARK LEWIS, M. D., Buffalo, N. Y.

[*American Journal of Obstetrics and Diseases of Women and Children*, November, 1907.]

IT is now almost a quarter of a century since Carl S. F. Credé of Liepsic, presented to the medical profession in an epoch-making paper, a discovery of such tremendous importance that

1. An address delivered by invitation at the twentieth annual meeting of the American Association of Obstetricians and Gynecologists, held at Detroit, September, 17-19 1907

its value has not been fully realized up to the present day. In the *Archive für Gynecologie* of October, 1883, Credé announced that by allowing a single drop of a 2 per cent. solution of nitrate of silver to flow from the end of a glass rod one-eighth of an inch in diameter upon the cornea of a newborn child, he had reduced the infections causing ophthalmia neonatorum in his great obstetrical clinic from 10 per cent. of the total number of births to two-tenths of 1 per cent. The importance of this announcement will be better understood when it is borne in mind that at that time, when many of us were students or young practitioners, the new science of medicine had but just been born. It was only four years before that Neisser had isolated the gonococcus, and I remember very well strangling in a carbolised atmosphere in an endeavor to see Billroth apply the principles of Lister. Your distinguished president—then a young man—had but just written that inspiring little monograph, which, though overshadowed since by his greater work, nevertheless served as an inspiration to thousands of young American surgeons "How We Treat Wounds To-day," and the whole medical world was awakened and had become keenly alive to the importance of microbial infections.

An additional importance was given to this great discovery by reason of the frightful virulence of the infection which it was found to control. The ugly, deformed, staphylomatous eyeballs, resulting from the corneal ulcerations of this disease, were seen everywhere. With the exception of smallpox, no single infection had begun to compare in the virulence of its results with ophthalmia neonatorum. Fully one-quarter of all of the pupils in the asylums for the blind were victims of this malady, and when, therefore, the statement was made by one of the most careful and dependable obstetricians in Europe that, by this absurdly simple precautionary measure, the child's chances of escaping the infection were increased fifty times, then obstetricians everywhere began repeating the experiments of Credé, and from enormous numbers upon which to base their statistics obtained singularly uniform results.

From tables published by Kostling of Halle, in 17,767 births with no treatment, 9.2 per cent. developed the ophthalmia of infancy, while in 24,723 births, in which the prophylactic treatment of the 2 per cent. nitrate of silver was employed, the infection developed in 0.65 per cent. In 4,000 births at the Sloan Maternity Hospital in New York during a period of six years, in which Credé's method was employed, not one case of ophthalmia developed. Later, in 1886, Credé reported 1,211 births, with 3 but slightly affected, or 0.25 per cent.

Other microbicides were tested for their prophylactic virtues —carbolic acid, iodoform, bichloride of mercury, the newer silver salts, protargol, argyrol, and even simple lavage with plain water. All had their advocates, and statistics were published galore. Indeed, a distinctive literature has appeared, so popular has the subject become, which if assembled would make large volumes. Meantime, too, scientific medicine had not been quiescent in other directions. We had learned how to drive yellow fever from its noisome haunts. We had hunted the typhoid germ to its polluted source. We knew under what conditions the bacillus of tuberculosis flourished and under which it faded away. Then, of course, in the case of a superficial infection like that which produces ophthalmia neonatorum, the nature and time of entry of which have been perfectly understood for more than two score of years, and which prophylaxis places almost absolutely under control, the disease must be practically wiped out of the land, and its ravages seen no more forever.

Let us see. The City of Buffalo is perhaps one of the most fortunate sections of the United States in the protection which it legally affords the new born child. For fourteen years it has had a law requiring midwives to attain a certain standard of proficiency in their work, this to be determined by a board composed of capable and reputable physicians. There exists also a law that every midwife shall at once report to some physician the existence of a case of ophthalmia neonatorum when it becomes known to her, under penalty of a fine. The Health Commissioner, Dr. Ernest Wende, has the deserved reputation of being one of the most capable and efficient public servants in this work in the United States.

Some months ago Dr. Wende sent return post cards to each physician engaged in the practice of obstetrics, to each midwife in the City of Buffalo, and to the superintendent of each hospital receiving lying-in cases. On each card was a request that a statement be made as to the number of cases of ophthalmia of the newborn that had occurred in the practice of the person receiving the card or in the institution during the previous year; what, if any, prophylactic had been used, and with what result. The total number of births in the City of Buffalo during the previous year had been 8,500. The returns did not include hospitals or other lying-in institutions in which, as Hubbell has shown, the frequency is much greater than in private practice. The total number of cases reported was one hundred and two, and if we add the unreported cases it will undoubtedly show ten times as many infections as should have occurred had adequate prophylaxis been employed.

"But," said the editor of a rather prominent southern journal shortly after the appointment of a committee on ophthalmia neonatorum by the president of the American Medical Association at its meeting at Boston in 1906, "does it not seem strange that legislation should be considered necessary to urge physicians and midwives to do their duty? We question very much that blindness due to ophthalmia will become much reduced by such legislation. The disease is certainly much less dreaded now than formerly, and Credé's method is more generally used; consequently, an additional legislative act seems an unnecessary burden on the statute books."

Is this disease, then, well understood and under control, and is the method of Credé generally and correctly employed? The secretary of your Association, who is president of the New York Board of Medical Examiners, refused recently to allow a medical applicant to qualify because, among other errors of knowledge or judgment, he advised as a prophylactic for ophthalmia neonatorum the 1 per cent. or 2 per cent. solution of nitrate of silver dropped in the eyes every 15 to 30 minutes. An examiner of undoubted ability and broad experience demurred at this ruling, saying that the remedy was right and the dosage was correct—the error was in its too frequent application, asking whether nature did not take care of such cases and whether disaster might be apprehended in consequence.

"I have been astonished," writes Dr. de Schweinitz, "in this comparatively enlightened age to find the appalling practices which go on among the poor in the Italian, negro, and other quarters of the city. It would seem to me that there is not any foolish thing that some equally foolish midwife will not put into the eyes of a new born baby provided there is any irritation. I will not burden this letter," he continues, "by citing instances of the appalling ignorance of certain physicians in regard to the proper method of employing silver under these circumstances, except to quote a case recently seen, where a physician used a 2 per cent. solution of nitrate of silver—a drop in each eye of a new born babe three times a day for three days, when there was not the slightest reason for its use, except a small discharge at the inner angles of the eyes totally free from the presence of gonococci. As a result the baby has a large white scar over one cornea and a smaller one over the other."

But, you urge, the standard of medical knowledge has been raised so much during the last twenty-four years that even if infections do occur, notwithstanding the neglect or mismanagement of a gonococcic or other microbial conjunctivitis in an infant, the resulting blindness or serious injury to the eyes must be an exceedingly rare occurrence, and it can no longer be a prolific cause of blindness. In the New York State School for the Blind children are received between the ages of five and twenty-one years. Many of those newly entering are of kindergarten age. Among those registered during the past four years—which shows how recent had been their affliction—26 per cent. had their blindness assigned by careful examiners to ophthalmia neonatorum, while 39 of the 150 registered, or one-quarter of the whole number, had their eyes, and consequently their lives, offered as a sacrifice to this Moloch of ignorance and neglect.

Philadelphia is certainly one of the most enlightened medical centers in the world. At Overbrook, one of its suburbs, is the school for the blind, concerning which Professor Allen, for many years its superintendent, writes me: "It happens that I have by me the main causes of blindness in percentages of the 536 pupils during my administration—or between 1890 and 1906 inclusive—a period of sixteen years. In this table ophthalmia neonatorum figures 29 per cent. of the whole. These cases were assigned by our own oculist," he writes, "Dr. George C. Harlan, and I think there is no mistake in the compilation of the averages."

Recently Mr. Simeon Snell, in a communication to the British Medical Association, reported that of 333 inmates of the Sheffield school for the blind, 136, or 42.36 per cent., had been blinded by ophthalmia neonatorum. These startling figures led to the unanimous passage of a resolution, proposed by Mr. Stevenson and seconded by Dr. Karl Grossman, that in the opinion of the section on ophthalmology the time had come for the British Medical Association to take action toward the prevention of ophthalmia neonatorum.

While I am not prepared to admit that our results are quite as bad as these figures would indicate—for in our schools for the blind are only the young, among whom the victims of ophthalmia neonatorum are always much the more numerous—we still must admit with humiliation and chagrin that the lesson which Credé taught has not been adequately learned. We have followed false gods in seeking easier methods, and our asylums are full of needless victims. But are all of these cases of blindness occurring in the schools of recent origin? Is it not possible that these blind children in the schools are the product of earlier negligence, and that more modern methods are now in use?

In the State of New York, as determined by the special commission, there were found to be 6,200 blind persons. Of these 569 were under one year of age, and under four years, including those under one year, were 959 children. In the State of Massachusetts, among 3,306 blind registered, 661, or more than 20 per cent., had become blind before their fifth year. If we exclude ulcerative conditions, due to bad hygiene and insufficient nourishment, which ought to be controllable, and congenital blindness, which in many instances can be avoided by preventing the congenitally blind from mating, we may safely assume that one-half of this number, or 10 per cent. of the whole, have given their eyes as a tribute to ignorance or neglect.

Such statements as these must give us pause. What do they mean? It would seem that while we have been combating more spectacular maladies—bringing our sanitary batteries to bear on typhoid, yellow fever, and tuberculosis—this elusive, if no less vicious malady, has in some degree at least escaped us. It is endemic but sporadic. It occurs only in from one in fifty to one in two hundred cases. The busy general practitioner, chiefly occupied with the better classes of society, may not see a case in years; meanwhile, he forgets its virulence and malignancy, and when his attention is called to the red, swollen, suppurating eyes of the baby—the mother being already convalescent—he thinks that any intelligent physician is capable of treating a conjunctivitis; indeed, he has been told so by the editor of one of our medical journals, and he uses *secundum artem* as he thinks a 2 per cent. solution of silver nitrate every two hours or a 10 per cent. solution of argyrol once a day. When, finally, the cornea sloughs, the curtain falls, and the light of those baby eyes is extinguished forever, he honestly believes that the virulence of the attack was such that no skill could have averted disaster. "He done his damndest. Angels could do no more."

But it is not chiefly at us of the medical profession that the accusing finger can with justice be pointed except for our sins of omission for responsibility not sustained. The negligence, the ignorance, the indifference concerning these conditions, find their apotheosis in the midwife. Permit me, if you please, to quote from a careful study of this subject made by a trained nurse, F. Elizabeth Crowell, on the "Midwives of New York," which appeared in *Charities and the Commons* for January 12 of the current year, and which will stand doubtless for the same class, whether they appear in Oakland or Oklahoma. Of one Italian midwife it is written, "her home was of the dirtiest, the condition of her hands was indescribable, her clothing was filthy, and her bag beggared description." As to the midwives' homes, 106 were absolutely filthy, as were the clothing and person of the midwife herself. As for the bags and their equipment from a professional standpoint, by far the greater number would make fit decorations for a chamber of horrors. Rusty scissors, dirty string, a bit of cotton, a few corrosive sublimate tablets, old rags and papers, some ergot and vaseline, a gummed catheter wired, were the usual contents.

Please God, these conditions will have been bettered by the recent law passed for Greater New York, putting these women under the control of the Health Department, for there are between nine hundred and one thousand of them (many, indeed, well trained, cleanly and intelligent) who were present during the past year at the confinement of 43,834 mothers in the metropolis—about 42 per cent. of the whole number of births. In the City of Buffalo, with a population of nearly 400,000, at about half of the births a midwife presides. In all probability, a like proportion obtains in every community in which the population is largely foreign. With regard to the prevalence of ophthalmia neonatorum there are no available statistics for New York City. The provision of the sanitary code regarding the reporting of contagious diseases to the Board of Health is practically a dead letter in connection with this particular disease.

What, then, is to be done? The control of the conditions producing this vast amount of unnecessary blindness is entirely possible and practical. Two factors are essential: first, more enlightenment—a broader, more general, popular knowledge of the causes and measures to be employed for its prevention; second, perfectly organised and coördinated effort in securing its control. The first is dependent upon the second. It is to us of the medical profession that the intelligent laity is looking for advice and instruction. Already societies for improving the condition of the blind and the prevention of blindness are asking what they can do to remedy the evil, and they can take no step—they can advance no movement—except as they have the authority and support of those who know what to do and how it should be done. Said that wonderful young woman, Helen Keller, a few days ago at a great meeting at Boston, speaking of blindness due to ophthalmia neonatorum: "The problem of prevention should be dealt with frankly. Physicians should take pains to disseminate knowledge needful for a clear understanding of the causes of blindness. The time for hinting at unpleasant truths is past. Let us insist that the States put into practice every known and approved method of prevention and that physicians and teachers open wide the doors of knowledge for the people to enter in. The facts are not agreeable reading. Often they are revolting. But it is better that our sensibilities should be shocked than that we should be ignorant of facts upon which rest sight, hearing, intelligence, morals, and the life of the children of men. Let us do our best to rend the thick curtain with which society is hiding its eyes from unpleasant but needful truths."

Said Dr. Juan Santos Fernandez, the distinguished Cuban ophthalmologist, and an honorary Fellow of this Association, touching upon this subject: "The important thing is to bring before the public mind, by means of constant propaganda, a knowledge of the danger to a recently born child, who is at all affected as to the eyes, the great harm which a husband affected with gonorrhea may cause his wife or offspring, and, side by side with these, to call the attention of the family to the facilities which the authorities will furnish them to guard against blindness. "This," he continues, "would be worth much more than penalties, and if there were a physician paid by the State (and in every county in the United States may such a health officer be found) to attend to the poor children affected, or to prevent their becoming affected, and this fact were to become known to the poor, they would surely seek his assistance, and he could fulfil his duties."

The campaign of education must be conducted along two distinct lines. As Stephenson has shown in his recent monograph on this subject, "It might be thought that medical practitioners needed no instruction as to the ways of preventing ophthalmia neonatorum, and doubtless the majority do not! There remains, however, the significnt fact that all of the babies who subsequently develop ophthalmia have not been delivered by midwives or uninstructed women," and Treacher Collins supplements this by saying: "Sad to relate, cases in which delay in the application of appropriate treatment has resulted in permanent damage are met with, where the mothers have been attended by a duly qualified medical man, and not by an ignorant midwife." It would seem to be essential, then, that exact information be conveyed to the members of the medical profession as to the manner in which the toilet of the infant should be performed—how the eyes should be cared for in order that they may be protected from infection.

Said Dr. W. O. Moore, in the *Medical Record*, nearly a quarter of a century ago, "if the physician would attend to the first bath of the new born and not leave the entire charge to a nurse,—perhaps an ignorant one,—much trouble and suffering might be averted." Schirmer speaks of this first bath as "giftwasser"—poison water—and simply dries the child's face, postponing the bath till the following day. Snell reports an almost absolute freedom from ophthalmia in the Jersop Maternity Hospital, simply by reason of exceeding care given to the toilet of the baby's eyes. "The patients," says Dr. Snell, "are among the poorest; some are inmates of the hospital, but the great majority are confined in their own homes. The midwives have received instructions that immediately the head is born, attention must be directed to the baby's eyes. Then with little pieces of lint moistened in tepid water the eyes are carefully washed, as well as the eyelids and parts adjoining. Subsequently in washing the child, care is taken to guard against reinfection. During the last three years there have been 2,242 labors among the in-patients and out-patients. In the first 200 there were a few cases of purulent ophthalmia, but in the last 2,000, since the method has been systematically adopted, not a single case occurred. Directions were also given to the nurses that if a child's eyes looked red it was to be taken at once to the hospital for a drop of nitrate of silver solution to be dropped into the eye." The plan depends for its efficacy on simple cleansing, and its success seems to be well worthy of note.

If these cases can be improperly handled by trained medical men, what must be done by midwives? Before these women can be instructed they must be known, registered, and made responsible. It seems quite impracticable to eliminate them entirely, but they must be made to show certain qualifications,—a certain amount of training, of fitness, of cleanliness, and decency. This means an organised health movement for the passage of State laws placing these irregular and limited practitioners under the control of the health authorities. They should be taught by lectures and simply but carefully worded instructions in their own language, how the toilet of the child should be conducted. In a circular issued by the Valentin Haüy Society for the Blind in Paris for distribution to midwives and mothers—and which is by far the best published, advice is given to the mother as to her personal care before and up to the time of the birth of the child, the precautions are detailed that should be taken to prevent ophthalmia, and the necessity is urged in black letter type of immediately seeking medical aid should the eyes of the child become at all inflamed. It cannot be hoped, however, even by the exercise of the greatest care to prevent the infection of every baby's eyes, although, as Stephenson says, with rigid care ophthalmia is brought almost to the vanishing point. The necessity for a prophylactic is emphasised.

Objection has been made to the classic Credé method because in a very few instances among the many thousands in which it has been employed excessive reaction has followed. The consensus of opinion seems to be, therefore, among obstetricians and ophthalmologists alike that while Credé's method properly employed is entirely safe and most effective in the hands of the trained accoucheur it might, if incorrectly used, give rise to undue irritation and it is advised, therefore, that the 1 per cent. solution of nitrate of silver, which is absolutely free from any danger to the eyes whatever and which does not produce silver catarrh, should in preference be employed by unskilled hands; but whatever prophylactic is used it should be prepared and gratutiously distributed by the health department. It should be enclosed in hermetically sealed and light-proof tubes or ampoules and the filing of the birth certificate which should be invariably required, would give the desired opportunity of placing the prophylactic in the hands of the accoucheur or midwife. The silver solution would then always be ready for use, would be of known strength and purity, of trivial cost and of incalculable value. A physician in Buffalo, whose routine practice was to use the Credé solution, omitted it twice in the course of a year because he did not happen to have a preparation of the silver in his bag. In both of these ophthalmia developed. Had these tubes been available half a dozen of them might be carried at once as the solution would be permanently stable and effective and two children would have escaped a danger which might have cost them their eyes.

The certainty too that the solution is of assured strength and purity when accuracy of dosage is of such great importance, must give added confidence in its use. In one reported case the error of a pharmacist made the preparation 20 per cent. of silver nitrate instead of the 2 per cent. called for, to the consternation of the doctor using it. The ampoules would prevent such errors.

If the midwife is to be held responsible for her neglect to use proper prophylactic measures under penalty of losing her license, as she should be, then she should have the prophylactic put in her hands with fullest directions for its use, that no excuse may exist for omitting it. This measure, which is of first importance, received the unqualified endorsement of Dr. Sidney Stephenson in his admirable monograph on ophthalmia neonatorum, in which he makes it one of the measures recommended for the control of this disease and concerning which he says: "At present in England we do something of the kind with regard to calf lymph and antitoxin. The principle, therefore, already is conceded."

It does not seem practicable to put ophthalmia neonatorum on the list of communicable diseases. Considering the fact that it is so frequently of gonorrheal origin, many physicians feel that to report it, with the name of the parents, would be a breach of professional confidence, but for the health officer to take a semiannual or annual canvass of the number of cases occurring in the practice of the physicians, midwives, and institutions of the locality, together with a statement of what, if any, prophylactic was used, with the resulting condition of the eyes in each instance, has a double value—namely, in serving to impress upon each one receiving the card the need of prophylaxis; and in obtaining statistics from which important conclusions may be drawn. It affords an opportunity, moreover, of conveying information to accoucheurs—that often may not, and soon would not, be necessary—but, meanwhile, it might be instrumental in saving eyes that would otherwise be lost.

The conclusions, in detail then, which are suggested are:

1. To secure the enactment of laws in each State or Federal territory placing the supervisory control and licensure of midwives with the Boards of Health; requiring that these unqualified practitioners be examined and registered in each county and that they be required to immediately report each case of ophthalmia occurring in their practice under penalty, if found guilty, of forfeiture of their license and a fine.

2. Distribution by health boards of circulars of advice to midwives and mothers giving instruction as to the dangers, method of infection, and prophylaxis of ophthalmia neonatorum.

3. The preparation and distribution by health boards of ampoules or tubes containing the chosen prophylactic. For midwives 1 per cent. solution of nitrate of silver is almost universally recommended by obstetricians and ophthalmologists. For physicians the Credé solution should consist of a 2 per cent. solution of chemically pure fused nitrate of silver. If used as directed by Credé, one drop from a glass rod ⅛ of an inch in diameter, it is free from excessive irritation and absolutely safe. To insure purity of the drug and accuracy of dosage the Credé solution should be given freely to physicians who make application therefor. This, however, should be merely advisory. The health department should be free to use such prophylactic as it may deem best.

4. Periodic report to Boards of Health by all physicians engaged in obstetrics of the number of cases of ophthalmia neonatorum that has occurred in their practice, whether or not a prophylactic was used—if so, what—together with the result.

5. The accomplishment of these measures by the appointment of committees through the various state and county societies whose coöperation would make concerted action possible.

6. To secure these ends the requested coöperation of the American Association of Obstetricians and Gynecologists, the Academy of Ophthalmology and Oto-Laryngology, the American Ophthalmological Society, the American Public Health Association, and such other organisations as may appoint committees on ophthalmia neonatorum.

If this plan of campaign be agreed upon with such modifications as obstetricians, ophthalmologists, and sanitarians may suggest, then a united and coördinated effort should be made to carry it into effect. If we would protect the babies—future citizens of the United States—from the poverty and misery of needless blindness, we must join hands and form a cordon reaching from Maine to Alaska and from the Great Lakes to the Gulf. The machinery is already in existence. It is but to act.

To what nobler work could the splendid organisation of the American Medical Association lend itself than in furthering such a cause. Such an organised and concerted movement steadily and effectively at work throughout the length and breadth of the land, would mark a new era in which the sodality of medicine would become the chief factor in a social uplift. It would bind the fraternity together with closer ties in an effort to shield humanity from its own follies and frailties. It would practically abolish ophthalmia as a cause of blindness, thereby saving millions to the commonwealth and immeasurably increasing the happiness and efficiency of humanity throughout the world.

454 Franklin Street.

